# Identification of the Aldo-Keto Reductase Responsible for d-Galacturonic Acid Conversion to l-Galactonate in *Saccharomyces cerevisiae*

**DOI:** 10.3390/jof7110914

**Published:** 2021-10-27

**Authors:** Dorthe Rippert, Federica Linguardo, Andreea Perpelea, Mathias Klein, Elke Nevoigt

**Affiliations:** Department of Life Sciences and Chemistry, Jacobs University Bremen gGmbH, 28759 Bremen, Germany; d.rippert@jacobs-university.de (D.R.); federica.linguardo@gmail.com (F.L.); a.perpelea@jacobs-university.de (A.P.); m.klein@jacobs-university.de (M.K.)

**Keywords:** d-galacturonic acid, l-galactonate, *Saccharomyces cerevisiae*, aldo-keto reductase, Gcy1

## Abstract

d-galacturonic acid (d-GalUA) is the main constituent of pectin, a complex polysaccharide abundant in several agro-industrial by-products such as sugar beet pulp or citrus peel. During several attempts to valorise d-GalUA by engineering the popular cell factory *Saccharomyces cerevisiae*, it became obvious that d-GalUA is, to a certain degree, converted to l-galactonate (l-GalA) by an endogenous enzymatic activity. The goal of the current work was to clarify the identity of the responsible enzyme(s). A protein homology search identified three NADPH-dependent unspecific aldo-keto reductases in baker’s yeast (encoded by *GCY1*, *YPR1* and *GRE3*) that show sequence similarities to known d-GalUA reductases from filamentous fungi. Characterization of the respective deletion mutants and an in vitro enzyme assay with a Gcy1 overproducing strain verified that Gcy1 is mainly responsible for the detectable reduction of d-GalUA to l-GalA.

## 1. Introduction

The sugar acid d-galacturonic acid (d-GalUA) is, apart from d-glucose and l-arabinose, an abundant monomer in pectins. Pectins are complex polysaccharides found in various types of plant cell walls [1]. Several agro-industrial waste streams such as sugar beet pulp or citrus peels are rich in pectins [2]. These carbohydrate-abundant residues have been considered attractive feedstocks for industrial biotechnology, particularly because they are pre-treated during food processing and virtually do not contain any lignin, making their hydrolysis relatively efficient [3,4]. The valorisation of pectin-rich biomass residues has to include concepts for the conversion of d-GalUA into valuable products since the sugar acid makes up approximately 70% of pectins [1,5,6].

**Figure 1:**
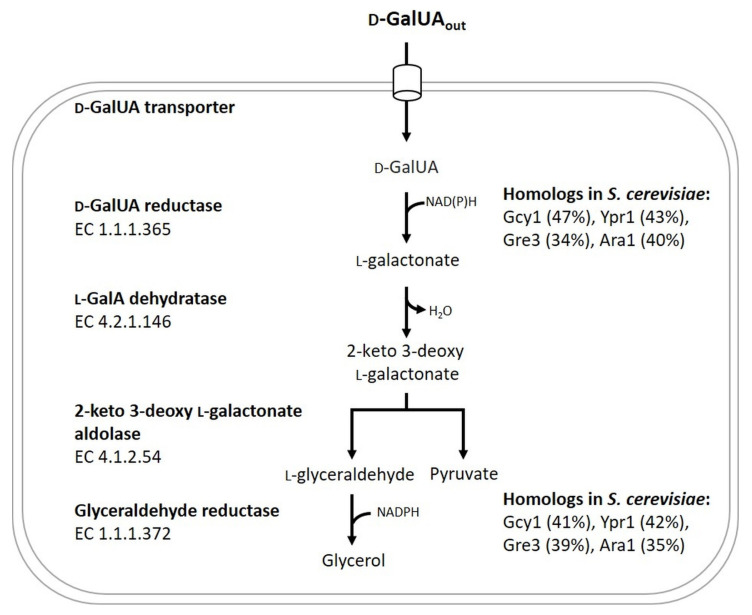
Overview of the enzymes comprising the d-galacturonic acid (d-GalUA) catabolic pathway commonly found in fungi able to utilize d-GalUA. In addition, *S. cerevisiae* proteins exhibiting a certain degree of homology to the fungal enzymes are shown. Percentage values in brackets indicate sequence identities between the respective yeast protein and the d-GalUA reductase Gar1 from *T. reesei* and the glyceraldehyde reductase Gld1 from *T. reesei*.

Certain bacteria and fungi are able to naturally utilize d-GalUA [7]. There are two known bacterial pathways for d-GalUA catabolism [7]. The pathway for d-GalUA utilization in fungal organisms seems to be conserved among asco- and basidiomycetes; it consists of four enzymes [8] and was first described in the mould *Trichoderma reesei* [9] (Figure 1). The products of these catabolic reactions are pyruvate and glycerol, which are subsequently further broken down in the cell’s central carbon metabolism. The first enzymatic step of the fungal d-GalUA pathway is the conversion of d-GalUA to l-galactonate (l-GalA) by a d-galacturonic acid reductase. l-GalA is further converted to 2-keto-3-deoxy-l-galactonate catalysed by an l-galactonate dehydratase. An aldolase splits the last intermediate into pyruvate and l-glyceraldehyde from which the latter compound is further converted to glycerol by an l-glyceraldehyde reductase [10,11].

With regard to the valorisation of pectin-rich biomass hydrolysates, fungal cell factories are generally preferred over bacteria due to a higher robustness towards the inhibitors present in such crude substrates. In fact, hydrolysates of pectin-rich biomass contain acetic acid and methanol from the acetylated and methyl-esterified polymers [5]. Both compounds are known as potent inhibitors of microbial growth and fermentation [5,12]. In hydrolysates of food waste streams, particularly acetic acid seems to reach concentrations which are inhibitory [13]. Last but not least, the abundant monomer d-GalUA is also a weak acid contributing to the potential stress in cells grown in such hydrolysates [14].

Among fungal organisms, baker’s yeast became an attractive organism in the context of valorising hydrolysates of pectin-rich biomass [15] even though it is neither able to naturally metabolize d-GalUA nor l-arabinose. Baker’s yeast is the organism of choice for the production of ethanol, and this has also been acknowledged in publications dealing with pectin-rich biomass [2,15,16]. Notably, the organism’s popularity has profited from the ease of metabolic engineering. By virtue of *S. cerevisiae*’s capacity for highly efficient homologous recombination, its accessibility to extensive targeted genetic modifications is extraordinary [17,18]. *S. cerevisiae* has already been successfully equipped with the ability to ferment pentose sugars to ethanol [19,20,21,22]. Notably, *S. cerevisiae* shows a relatively high tolerance to alcohols and organic acids, low pH and low oxygen levels [2,23,24,25,26]. These natural characteristics also contribute to the attractiveness of *S. cerevisiae* for valorising pectin-rich residues.

There have already been several attempts to implement a heterologous d-GalUA pathway in *S. cerevisiae* [11,27,28]. However, the achieved rates of d-GalUA consumption were far from economically viable even under aerobic conditions and in the presence of a co-substrate. Recently, our laboratory significantly improved the situation by constructing a *S. cerevisiae* strain able to consume d-GalUA with a specific rate of 0.23 g g_CDW_^−1^ h^−1^ (CDW = cell dry weight) [29]. A challenging aspect when considering homo-ethanol fermentation from pectin hydrolysates is the electron balance since d-GalUA is more oxidized than neutral sugars. In this context, we have considered glycerol an ideal co-substrate as a donor of electrons and embarked on the challenge to establish co-fermentation of d-GalUA and glycerol. In our study [29], a fungal d-GalUA pathway was inserted in a strain of the yeast *S. cerevisiae* previously equipped with an efficient NAD-dependent glycerol catabolic pathway. The study delivers the proof of concept for a co-fermentation of the two ‘respiratory’ carbon sources to ethanol and demonstrates a fast and complete consumption of d-GalUA in crude sugar beet pulp hydrolysate, at least under aerobic conditions [29].

It has been an interesting auxiliary result of the study conducted by Perpelea et al. [29] that the reference strain (without any heterologous d-GalUA transporter or catabolic enzyme) also consumed a certain amount of d-GalUA. It also became obvious that this conversion depended on the presence of the co-substrate glycerol. This result matched reports of other authors who previously showed that (i) some d-GalUA can be taken up by one or more endogenous uptake mechanism(s) in *S. cerevisiae* [27,30] and (ii) one or more endogenous reductase(s) in *S. cerevisiae* can convert d-GalUA to l-GalA. This enzyme reaction corresponds to the first step of the fungal d-GalUA pathway [31,32]. The goal of the current study was to identify the enzyme(s) that is/are responsible for the observed conversion of d-GalUA to l-GalA in baker’s yeast.

## 2. Materials & Methods

### 2.1. Strains, Medium Composition and General Cultivation Conditions

All strains and plasmids used in this study are listed in Table 1 and Table S1, respectively. Yeast cells were routinely grown on solid YPD medium containing 10 g L^−1^ yeast extract, 20 g L^−1^ peptone, 20 g L^−1^ glucose and 15 g L^−1^ agar. For yeast cultivation, the agar plates were placed in a static incubator at 30 °C. In case of selection of transformed strains, media were supplemented with 20 µL mL^−1^ phleomycin.

*E. coli* DH5α was used for plasmid maintenance and isolation. *E. coli* strains carrying plasmids were routinely grown at 250 rpm and 37 °C in lysogeny broth containing 10 g L^−1^ peptone, 5 g L^−1^ yeast extract, 10 g L^−1^ NaCl (pH 7.0) and 100 mg L^−1^ ampicillin for selection. Plasmids were isolated by using the GeneJET^TM^ Plasmid Miniprep Kit (Thermo Fisher Scientific, Waltham, MA, USA).

### 2.2. General Molecular Biology Techniques

Preparative PCRs for cloning were performed using Phusion^®^ High-Fidelity DNA Polymerase (New England Biolabs, Frankfurt am Main, Germany). PCR conditions were adapted according to the guidelines of the manufacturer. PCR products were purified using the GeneJET^TM^ PCR Purification Kit (Thermo Fisher Scientific). Transformation of *S. cerevisiae* was performed according to the lithium acetate method described by Gietz et al. [36].

### 2.3. Construction and Verification of Deletion Strains

For gene disruption, deletion cassettes containing the phleomycin (*ble*) resistance marker were amplified from pUG66 [37] using the primers listed in Table S2 (Supplementary Material). The primers used for amplification contained at their 5′-terminus a 40–50 bp sequence complementary to the region immediately upstream or downstream of the start or stop codon of the gene to be deleted. The cassettes were used to transform *S. cerevisiae* and their integration at the target locus by homologous recombination resulted in deletion of the respective genes. Proof of the correct integration of all deletion cassettes was performed by diagnostic PCR (primers listed in Table S2, Supplementary Material) using One*Taq* Quick-Load DNA Polymerase (New England Biolabs) according to the manufacturer’s guideline. Single cell colonies obtained after transformation were re-streaked on selective agar plates. Genomic DNA was isolated according to a modified protocol from Hoffman and Winston [38]. Approximately 50 mg of cells was suspended in 200 µL of TE buffer (10 mM Tris, 1 mM EDTA, pH 8.0). Afterwards, 300 mg of acid-washed glass beads (diameter of 0.425–0.6 mm) and 200 µL of phenol/chloroform/isoamyl alcohol (25:24:1) were added. The tubes were vortexed at a maximum speed for 2 min and centrifuged at 15,700 g for 10 min. From the aqueous phase, 1 µL was used as template in 20 µL PCR reactions.

### 2.4. Construction and Verification of the Reverse-Engineered Strain CEN.PK RE

The reverse-engineered strain CEN.PK RE is a derivate of the previously described strain CEN.PK113-1A *UBR2_CBS_* [33]. To construct the strain CEN.PK RE (Table 1), the endogenous *GUT1* allele of strain CEN.PK113-1A *UBR2_CBS_* was replaced by the *GUT1* allele from strain JL1 [35]. The only difference was a single point mutation within the *GUT1* expression cassette, as reported by Ho et al. [35]. This point mutation improves growth in synthetic glycerol medium. The allele replacement was achieved by using the same two-step *GIN11* counter-selectable strategy and primers described by Ho et al. [35].

### 2.5. Media for Analysing Glycerol and d-GalUA Utilization of Engineered S. cerevisiae Strains

All pre-cultures were cultured in synthetic medium containing 20 g L^−1^ glucose and ammonium sulphate as carbon and nitrogen source, respectively. All experiments for assessing glycerol and d-GalUA consumption in shake flask batch cultivation were performed in synthetic medium, containing 60 mL L^−1^ (75.6 g L^−1^) glycerol and 5 g L^−1^ d-GalUA (d-galacturonic acid sodium salt, Chemodex, St. Gallen, Switzerland) as carbon sources and urea as nitrogen source. The synthetic medium was prepared according to Verduyn et al. [39] and contained 3 g L^−1^ KH_2_PO_4_, 0.5 g L^−1^ MgSO_4_·7H_2_O, 15 mg L^−1^ EDTA, 4.5 mg L^−1^ ZnSO_4_·7H_2_O, 0.84 mg L^−1^ MnCl_2_·2H_2_O, 0.3 mg L^−1^ CoCl_2_·6H_2_O, 0.3 mg L^−1^ CuSO_4_·5H_2_O, 0.4 mg L^−1^ NaMoO_4_·2H_2_O, 4.5 mg L^−1^ CaCl_2_·2H_2_O, 3 mg L^−1^ FeSO_4_·7H_2_O, 1 mg L^−1^ H_3_BO_3_ and 0.1 mg L^-1^ KI. After heat sterilization of the salts, 1 mL L^−1^ of a vitamin stock solution was added, resulting in the following final concentrations: 0.05 mg L^−1^ d-(+)-biotin, 1 mg L^−1^ d-pantothenic acid hemicalcium salt, 1 mg L^−1^ nicotinic acid, 25 mg L^−1^ myo-inositol, 1 mg L^−1^ thiamine chloride hydrochloride, 1 mg L^−1^ pyridoxine hydrochloride and 0.2 mg L^−1^ 4-aminobenzoic acid. In case urea was used as the nitrogen source (in main culture media), an appropriate aliquot of a stock solution was added after autoclaving to obtain a final concentration of 2.8 g L^−1^, while in pre-cultures, 5 g L^−1^ ammonium sulphate was added before heat sterilization. The pH was adjusted to 6.5 with 4 M KOH for the synthetic glucose medium. The pH of the synthetic glycerol medium containing d-GalUA was adjusted to either 3.0 using 2 M H_3_PO_4_ or 5.0 using 4 M KOH.

### 2.6. Characterization of S. cerevisiae in Shake Flask Batch Cultivations

Cells from a single colony were used to inoculate 3 mL of synthetic glucose medium in a 10 mL glass tube and were incubated at orbital shaking of 200 rpm and 30 °C for 16 h. The pre-culture was used to inoculate 10 mL of the same medium in a 100 mL Erlenmeyer flask, adjusting an OD_600_ of 0.2. This culture, hereafter referred to as intermediate culture, was cultivated under the same conditions for 24 h. The appropriate culture volume from the intermediate culture (in order to later adjust an OD_600_ of 0.2 in 50 mL or 100 mL of synthetic glycerol medium containing d-GalUA) was centrifuged at 800 g for 5 min. The cell pellet was then washed once by re-suspending the cells in synthetic glycerol medium containing d-GalUA. The cell suspension was centrifuged again and re-suspended in 50 mL or 100 mL of the same medium in a 500 mL Erlenmeyer flask. The main cultures were incubated at orbital shaking of 200 rpm and 30 °C, and samples for OD_600_ determination and HPLC analysis were taken at regular time intervals.

### 2.7. Metabolite Analysis by HPLC

The samples (1.0 mL culture supernatant) were filtered through 0.2 μm Minisart RC membrane filters (Sartorius, Göttingen, Germany) and stored at −20 °C until analysis. Detection and quantification of glycerol and d-GalUA was performed using a Waters HPLC system (Eschborn, Germany) consisting of a binary pump (Waters 1525), an injector system (Waters 2707), a Waters column heater module WAT038040 and a refractive index detector (Waters 2414). An Aminex HPX-87H cation exchange column (Bio-Rad, München, Germany) coupled to a Micro-guard*^®^* cation exchange column (Bio-Rad) was used for chromatography. As an eluent, 5 mM H_2_SO_4_ at a flow rate of 0.6 mL min^−1^ was used. The column was kept at 45 °C. A sample volume of 20 μL was injected. Under these conditions, the retention times were about 14 min for glycerol and 8.5 min for d-GalUA. l-GalA was detected under the same conditions. Notably, l-GalA has not been commercially available and the l-GalA used here as a standard was obtained by hydrolysis of l-galactono-1,4-lactone (Sigma-Aldrich, Schnelldorf, Germany) (titrating the solution to pH 8 with NaOH) and was kindly provided by Peter Richard (VTT, Espoo, Finland). This self-made standard resulted in two peaks with retention times of 9.3 and 9.7 min. Data were analysed using the Breeze 2 software (Waters).

### 2.8. Selectable Genetic Marker Replacement in Multicopy Plasmids for GCY1 Overexpression

The construction of a 2*µ*-based plasmid p424GCY1 for the overexpression of GCY1 has been described in Nguyen and Nevoigt [40]. This plasmid and the corresponding empty vector p424GPD [41] contain the *TRP1* gene as the selectable genetic marker. The auxotrophic marker was replaced by the dominant marker conferring resistance to phleomycin in order to be used in prototrophic *S. cerevisiae* strains. The *ble* (from bacterial transposon Tn5) cassette was amplified from pUG66 [36] using primers listed in Table S3 (Supplementary Material). The PCR product and the respective plasmid were used to transform *S. cerevisiae*, and the marker replacement occurred via in vivo recombination. The correct marker replacement was checked via diagnostic PCR using the primers listed in Table S3 (Supplementary Material) and OneTaq Quick-Load DNA Polymerase (New England Biolabs) according to the manufacturer’s guideline. The correct transformants were used for the d-GalUA reductase enzyme assay.

### 2.9. In Vitro Measurement of d-GalUA Reductase Activity

Cell disruption and enzyme assay were carried out following the procedure described by Biz et al. [11] with slight modifications. Yeast cells were grown overnight in 50 mL liquid YPD medium supplemented with 30 µg/mL of the antibiotic phleomycin. A culture volume of 50 mL with an OD_600_ of 5 (corresponding to ca. 120 mg CDW) was harvested. To ensure harvesting of the same number of cells in cultures of different optical densities, the appropriate culture volume was calculated based on the actual OD_600_ of the culture. Cells were washed once with 50 mL water and re-suspended in 1 mL 50 mM sodium phosphate buffer (pH 7) with addition of the EDTA-free Protease inhibitor cocktail cOmplete (Roche, Basel, Switzerland) according to the manufacturer’s guideline, and 0.5 g glass beads (0.4 mm diameter) was added. Cells were disrupted by vigorous vortexing for 10 min at 4 °C using Genie 2 Vortex Mixer (Scientific Industries, New York, USA) equipped with an adapter holding for 1.5 mL tubes. The samples were centrifuged for 15 min at 16000*g* and 4 °C to remove glass beads and cell debris. The enzyme activity was assayed in the supernatant by recording the decrease in absorbance at 340 nm caused by the oxidation of NADPH in a mixture containing 100 mM sodium phosphate buffer (pH 7), 0.2 mM NADPH, 10 mM d-GalUA, and 50 µL crude yeast extract in a total volume of 1 mL. After incubating the mixture without d-GalUA at 30 °C for 1 min, the reaction was started by the addition of 100 µL of 100 mM d-GalUA. Protein concentration in crude cell extracts was determined by the BCA assay [42] using a kit from Thermo Scientific.

## 3. Results

### 3.1. The S. cerevisiae Derivate CEN.PK113-1A Equipped with the NAD-Dependent DHA Pathway for Glycerol Utilization Was Able to Convert d-GalUA to l-GalA

As mentioned in the introduction, a glycerol-utilizing derivative of the well-characterized *S. cerevisiae* strain CEN.PK consumed some d-GalUA when added to synthetic glycerol medium [29]. In the respective strain referred to here as CEN.PK DHA [34], the endogenous so-called l-glycerol-3-phosphate (l-G3P) pathway for glycerol catabolism (reviewed in Klein et al. [43]) was replaced by the NAD-dependent dihydroxyacetone (DHA) pathway [44]. Moreover, the strain carried an expression cassette for a heterologous aquaglyceroporin from *Cyberlindnera jadinii* for improved glycerol uptake [45] and a replacement of the native *UBR2* allele by the respective allele from the natural *S. cerevisiae* isolate CBS 6412-13A [33]. The *UBR2* gene encodes a cytoplasmic ubiquitin-protein ligase identified in the study of Swinnen et al. [33]. The authors focused on a QTL analysis to identify the crucial mutations allowing the natural isolate CBS 6412-13A to grow in synthetic glycerol medium in contrast to the popular laboratory strain CEN.PK113-1A. One of the crucial genetic determinants was the sequence of the *UBR2* gene. Swinnen et al. [33] demonstrated that the endogenous *UBR2* allele of CEN.PK113-1A encodes for a truncated version of the Ubr2 protein. Moreover, the authors demonstrated that the replacement of the endogenous *UBR2* allele by the allele from strain CBS 6412-13A is essential for establishing growth in synthetic glycerol medium in CEN.PK strains. The combination of the above-mentioned modifications in the strain CEN.PK DHA allow the strain to grow in synthetic glycerol medium with a maximum specific growth rate of 0.24 h^−1^ [34] while wild-type CEN.PK strains are unable to grow at all under these conditions [46].

**Figure 2:**
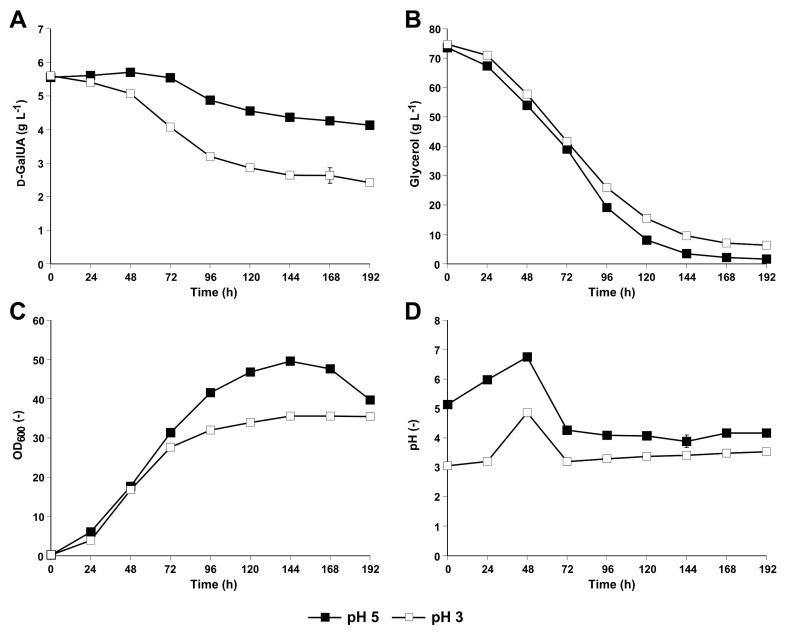
d-GalUA consumption profile of the strain CEN.PK DHA in synthetic glycerol medium supplemented with d-GalUA at an initial pH of 5 and 3. Cultivations were performed in 500 mL flasks containing 100 mL of synthetic medium with glycerol and d-GalUA with an initial pH of 5 (closed squares) or an initial pH of 3 (open squares) and urea as the source of nitrogen. Samples were taken in 24 h intervals, and culture supernatants were analysed by HPLC to follow the consumption of d-GalUA (**A**) and glycerol (**B**). Growth was recorded by determining the optical density of the cultures at a wavelength of 600 nm (OD_600_) (**C**). pH variation was measured over time (**D**). All mean values and standard deviations were derived from biological triplicates.

The finding of Perpelea et al. [29] that the strain CEN.PK DHA (Gly reference strain) utilizes some d-GalUA has been obtained in shake flask experiments conducted with an initial pH of 5. Here, we repeated the experiment at an initial pH of 3 since *S. cerevisiae* strains (without a dedicated d-GalUA transporter) have previously been reported to show a significantly faster natural uptake of d-GalUA at pH values below 3.51, which is the pK_a_ of d-GalUA [30]. A comparison of d-GalUA consumption of the CEN.PK DHA strain at initial pH values of 3 and 5 confirmed that d-GalUA consumption increased significantly at the lower pH (Figure 2). As a next step, we checked culture supernatants for the presence of l-GalA since Benz et al. [31] reported that a *S. cerevisiae* strain expressing a heterologous d-GalUA transporter (but no heterologous enzymes for d-GalUA catabolism) converted d-GalUA to l-GalA. Indeed, HPLC analysis of the culture supernatants obtained with our strain in the presence of d-GalUA revealed a decrease in the d-GalUA peak area. Moreover, the size of a peak with a retention time of ~9.7 min that was not visible in a control cultivation in synthetic glycerol medium without the addition of d-GalUA increased over time. We assume that the peak corresponds to l-GalA (Material and Methods). We used LC-MS to verify the presence of l-GalA in the supernatants of the CEN.PK DHA culture grown for 144 h in synthetic glycerol medium supplemented with d-GalUA (initial pH 3).

### 3.2. Deletion of GCY1 Abolished Formation of l-GalA from d-GalUA

In order to identify candidate genes encoding the endogenous d-GalUA reducing activity in *S. cerevisiae*, we first searched for sequence similarities among baker’s yeast proteins to the best-known fungal d-GalUA reductases, i.e., GaaA from *Aspergillus niger* (NCBI Accession number: ABQ53587.1) [47] and Gar1 from *T. reesei* (NCBI Accession number: AAX54673.1) [48], by performing a BLAST (basic local alignment search tool) analysis. While this search did not reveal any yeast ortholog for *A. niger* GaaA, we identified the four proteins Gcy1 (NCBI Accession number: NP_014763.1), Ypr1 (NCBI Accession number: NP_010656.1), Gre3 (NCBI Accession number: NP_011972.1) and Ara1 (NCBI Accession number: NP_009707.3) from *S. cerevisiae* which show relatively high sequence identities to the *T. reesei* Gar1 (47%, 43%, 34% and 40%, respectively) (Figure 1). These proteins were previously characterized as aldo-keto reductases with a wide substrate spectrum [49,50]. Notably, we did not further analyse Ara1 since it prefers to catalyse the reaction in the direction of aldose oxidation, as shown by Kim et al. [51].

**Figure 3:**
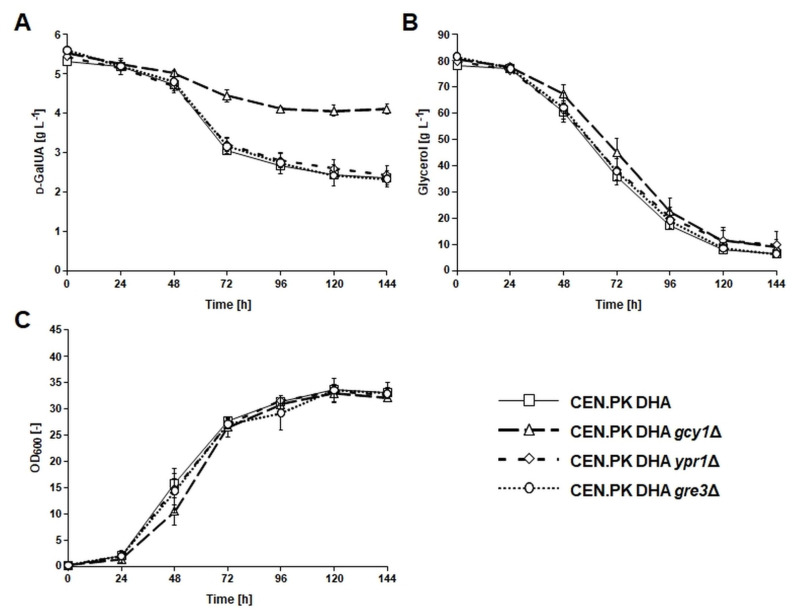
d-GalUA and glycerol utilization, as well as biomass formation of the strain CEN.PK DHA, and the impact of deleting the genes (*GCY1*, *YPR1* and *GRE3*) encoding unspecific aldo-keto reductases and showing homology to fungal enzymes involved in the d-GalUA catabolic pathway. Strains were cultivated in synthetic glycerol medium supplemented with d-GalUA and urea as the source of nitrogen. Cultivations were performed in 500 mL flasks containing 50 mL medium with an initial pH of 3. Samples were taken in 24 h intervals, and culture supernatants were analysed by HPLC to follow the consumption of d-GalUA (**A**) and glycerol (**B**). Growth was recorded by determining the optical density of the cultures at a wavelength of 600 nm (OD_600_) (**C**). All mean values and standard deviations were derived from biological triplicates.

**Figure 4:**
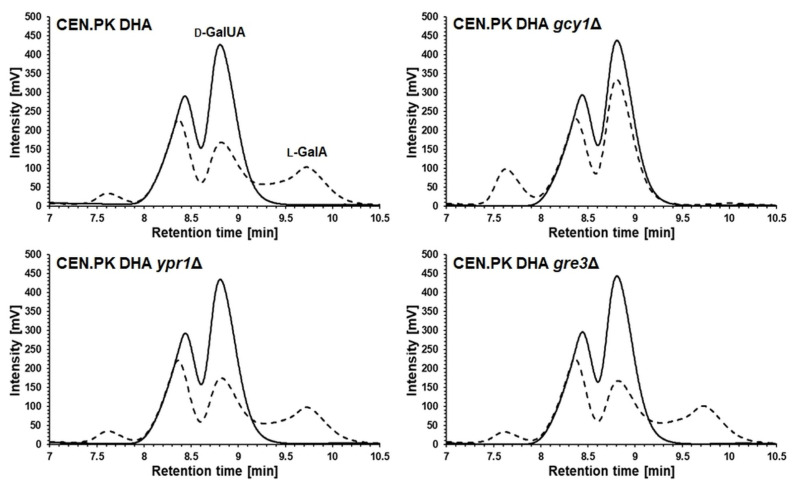
HPLC chromatograms of culture supernatants from shake flask cultivations in synthetic glycerol medium supplemented with d-GalUA conducted with the reference strain CEN.PK DHA and the three isogenic mutant strains in which *GCY1*, *GRE3* or *YPR1* (encoding unspecific aldo-keto reductases) was deleted. Cultivation conditions are described in the figure caption of Figure 3. d-GalUA and l-GalA were detected via the Waters 2412 RI detector. The retention time of d-GalUA was ~8.8 min, and the retention time of l-GalA was ~9.7 min. For each strain, a sample after 144 h (dashed line) was compared with a sample from the respective strain that was taken at the beginning of the batch cultivation in shake flasks (straight line).

To test the contribution of the three identified *S. cerevisiae* aldo-keto reductases in the conversion of d-GalUA to l-GalA in the strain CEN.PK DHA, single gene deletions of *GCY1*, *YPR1* and *GRE3* were constructed in this genetic background. The resulting deletion mutants were characterized in synthetic glycerol medium supplemented with d-GalUA. In accordance with the previous findings regarding natural d-GalUA uptake by *S. cerevisiae*, the initial pH of the medium was set to 3. The d-GalUA concentration in the cultivations of the *ypr1* and *gre3* deletion strains decreased with the same kinetics as observed for the reference strain (CEN.PK DHA). However, d-GalUA conversion was significantly reduced for the *gcy1*Δ mutant (Figure 3). In contrast, growth and glycerol utilization of all mutants remained unaffected. The resulting hypothesis that Gcy1 is the endogenous enzyme that converts d-GalUA to l-GalA was substantiated by the fact that no l-GalA peak was detectable in HPLC chromatograms of supernatants taken from the culture of the *gcy1*Δ mutant throughout the entire cultivation (Figure 4). The absence of the l-GalA peak in this strain was also confirmed for the culture taken after 144 h of cultivation via LC-MS. In contrast, a peak with increasing area for l-GalA was visible in the culture supernatants of the reference strain, the *ypr1* and the *gre3* deletion mutant (Figure 4). Interestingly, all HPLC chromatograms from samples taken after 144 h of cultivation revealed a peak of unknown identity (retention time ~7.7 min) whose area was significantly increased in the *gcy1*Δ mutant compared with all other strains (Figure 4). The appearance of the respective peak was dependent on the presence of d-GalUA in the medium. This was confirmed by cultivating the *gcy1*Δ mutant in the same synthetic glycerol medium but without d-GalUA.

### 3.3. Conversion of d-GalUA to l-GalA Was Not Dependent on the DHA Pathway for Glycerol Catabolism

*GCY1* has, in several independent studies, been demonstrated to encode a strictly NADPH-dependent enzyme [50,52,53,54]. We scrutinized whether Gcy1 merely provided the NADPH for the in vivo d-GalUA reduction while the actual reduction of d-GalUA was carried out by another endogenous enzyme. In this context, it becomes relevant to note that the *S. cerevisiae* strain CEN.PK DHA (used in the above experiments to delete *GCY1*, *YPR1* and *GRE3*, respectively) carries multiple genetic modifications, including the strong overexpression of *Opgdh*, which is a dehydrogenase (Figure S1B). These modifications allow the superior growth of this strain in synthetic glycerol medium. Although we considered it unlikely that the heterologous Opgdh in the respective strain would have a side activity towards reduction of d-GalUA, we decided to check whether the d-GalUA-reducing activity detected in the presence of glycerol in the strain CEN.PK DHA was dependent on the DHA pathway. Unfortunately, we could not use the corresponding wild-type CEN.PK113-1A strain (Figure S1A) since this strain cannot grow in synthetic glycerol medium at all [46] and the in vivo d-GalUA reducing activity can only be observed during co-consumption of glycerol. A solution was the use of a reverse-engineered derivative of the strain CEN.PK 113-1A. This strain—referred to here as CEN.PK RE (Table 1)—does not contain the DHA pathway but can still grow in synthetic glycerol medium with a moderate rate solely based on the l-G3P pathway (Figure S1C). The only genetic modifications in strain CEN.PK RE compared with the wild-type CEN.PK113-1A are the following allele replacements: (i) the endogenous *UBR2* allele by *UBR2* from strain CBS 6412-13A and (ii) the wild-type CEN.PK *GUT1* allele by the *GUT1* allele from JL1 [35]. When the strain CEN.PK RE was tested in synthetic glycerol medium supplemented with d-GalUA at an initial pH 3, it did not consume any d-GalUA before 144 h of cultivation (Supplementary Material, Figure S2A). It is assumed that this delay matches the significantly slower biomass formation accompanied by a reduced consumption of glycerol (compared with the strain CEN.PK DHA) (Supplementary Material, Figure S2B,C). Still, the HPLC chromatogram of the medium sample taken after 192 h of cultivation displayed a small l-GalA peak (Supplementary Material, Figure S2D). The low quantity fits to the comparatively low amount of d-GalUA consumed (Supplementary Material, Figure S2A). Thus, the results suggest that the existence of the endogenous d-GalUA reductase activity in *S. cerevisiae* is independent of the genetic modifications in connection to the DHA pathway. However, the more efficient glycerol consumption in the DHA pathway strain seemed to provide the required reducing power faster, thereby facilitating the conversion.

### 3.4. Overexpression of GCY1 Resulted in Increased In Vitro Conversion of d-GalUA

In order to verify that Gcy1 is directly responsible for the conversion of d-GalUA to l-GalA, we transformed the strains CEN.PK DHA and CEN.PK113-1A with a 2*µ*-based multicopy vector overexpressing *GCY1* (p424GCY1-ble, Table S1). The same two strains were transformed with the empty vector (p424GPD-ble, Table S1) to obtain the respective isogenic reference strains without *GCY1* overexpression. The in vitro measurement of d-GalUA reducing activity with NADPH as a co-factor detected a specific enzyme activity of 294 ± 44 mU/mg protein in crude cell extracts of the two *GCY1* overexpressing strains. In contrast, the negative controls with the empty vector did not reveal any enzyme activity under these conditions. Moreover, both strains did not show any d-GalUA reductase activity if NADH was used as co-factor. The results confirm that Gcy1 is able to reduce d-GalUA in an NADPH-dependent manner.

## 4. Discussion

In this study, we provide the experimental evidence that the aldo-keto reductase Gcy1 is mainly responsible for the slight enzymatic conversion of d-GalUA to l-GalA that has been observed when our *S. cerevisiae* strain solely equipped with the DHA pathway for glycerol catabolism was cultivated in a mixture of glycerol and d-GalUA [29]. Apart from the respective results obtained in our previous work (at initial pH 5) and in our current study (at initial pH of 3), several past reports of other authors also pointed to the ability of *S. cerevisiae* to take up d-GalUA and/or convert it to l-GalA. For example, l-GalA was intracellularly detected by means of LC-MS/MS in a strain solely carrying a heterologous transporter for d-GalUA uptake [31]. In another study, the authors expressed a d-GalUA reductase from *Cryptococcus diffluens* in *S. cerevisiae* in order to demonstrate the production of l-GalA from d-GalUA [32]. The respective reference strain (i.e., wild-type *S. cerevisiae* without any heterologous reductase) also produced a small amount of l-GalA from d-GalUA if galactose was used as co-substrate. In addition to the fact that *S. cerevisiae* exhibits a d-GalUA reducing activity, our current study also confirms that this organism can take up d-GalUA to a certain degree even at pH values higher than the pK_a_ of d-GalUA (3.51) [30]. Nevertheless, the observed d-GalUA consumption (related to biomass) detected in the current study was faster in the experiment conducted at the initial pH of 3, a result matching the assumption of a more efficient d-GalUA uptake at lower pH (Figure 2).

Based on the data generated in the study of Perpelea et al. [29], we know that d-GalAU is only consumed by the strain CEN.PK DHA if the co-substrate glycerol is added, which allows growth. It also seems that the enzymatic conversion of d-GalAU to l-GalA mainly occurs during the phase of the cultivation in which growth is most pronounced (Figure 3). Gcy1 has been shown to be strictly dependent on NADPH, and it is obvious that the redox equivalents have to be delivered during the metabolism of glycerol. It is likely that the NADPH is mainly formed during the oxidative pentose phosphate pathway and that the flux through this pathway is increased during growth [55].

Gcy1 was initially identified as a galactose-inducible crystalline-like protein (yeast homolog of eye-lens protein ρ-crystallin from European common frog) [56]. Because of its sequence identity to a peptide fragment of glycerol dehydrogenase from *A. niger,* it was originally speculated that Gcy1 might function as a GDH converting glycerol to DHA since Gcy1 expression was strongly increased upon osmo-stress [57,58]. In addition, the expression of *GCY1* was significantly higher in medium containing glycerol as the sole source of carbon, but not ethanol [59]. However, all efforts to measure GDH activity in vitro have failed so far [40,57]. Nonetheless, Zhang et al. [60] claimed an improved glycerol utilization in a *S. cerevisiae* strain in which the genes *GCY1* (encoding an NADPH-dependent aldo-keto reductase) and *DAK1* (encoding a dihydroxyacetone kinase) are overexpressed.

Gcy1 seems to be a relatively promiscuous enzyme with a broad substrate spectrum. Chang et al. [50] expressed the *GCY1* gene from *S. cerevisiae* in *E. coli*, and the activity of the gene product was tested with several substrates such as d,l-glyceraldehyde, ρ-nitrobenzaldehyde, benzaldehyde and phenylglyoxal. d,l-glyceraldehyde was the substrate with the highest k_cat_, and NADPH was shown to be the co-factor. Interestingly, *S. cerevisiae* Gcy1 shows high homology to different enzymes contributing to d-GalUA catabolism in filamentous fungi. It shares a high sequence homology with Gar1 from *T. reesei* (47%), *A. niger* [61] (51%) and *Botrytis cinerea* [62] (52%). Gar1 has been demonstrated to catalyse the first step of the d-GalUA catabolic pathway, i.e., d-GalUA to l-GalA [48]. The current study demonstrates for the first time that the baker’s yeast homologue Gcy1 also shows d-GalUA reducing activity. The fungal d-GalUA reductase Gar1 from *T. reesei* shows a certain flexibility for its substrate and is for example able to convert d,l-glyceraldehyde to glycerol and d-glucuronic acid (d-GlcUA) to l-gulonate [7,48], confirming the broad substrate spectrum of Gcy1 and its homologs. Second, Gcy1 also shows relatively high homology (41%) to the last enzyme of the fungal d-GalUA pathway, i.e., from d,l-glyceraldehyde to glycerol (Gld1 from *T. reesei* or GaaD from *A. niger*) (Figure 1) [47,63]. This matches the affinity of Gcy1 for d,l-glyceraldehyde (see above). Therefore, it is possible that enzymes of the EC number 1.1.1.365 and 1.1.1.156 share the same evolutionary origin and are all paralogs or orthologs.

Protzko et al. [27] already demonstrated that the presence of a heterologous l-glyceraldehyde reductase is not required for a fully functional d-GalUA pathway since glycerol accumulates in a strain equipped with just the first three enzymes of the fungal d-GalUA pathway. Based on these results, the authors postulated that *S. cerevisiae* has an endogenous enzyme that can take over the last reaction of the d-GalUA pathway and claimed that Ypr1 is most probably responsible for the conversion of d,l-glyceraldehyde to glycerol in *S. cerevisiae.* However, since Gcy1 is a paralog of Ypr1 and since it shows a high homology to the fungal l-glyceraldehyde reductase, one cannot exclude the possibility that this protein also contributes to this reaction.

Apart from the fact that Gcy1 is responsible for d-GalUA conversion to l-GalA in *S. cerevisiae*, our data suggest the existence of an additional enzyme in *S. cerevisiae* that is able to marginally convert d-GalUA to another product. In fact, the HPLC chromatogram of culture supernatants from the *gcy1* deletion strain revealed a peak of increased peak area and so far unknown identity. The appearance of the peak was dependent on the presence of d-GalUA in the culture medium. This peak of unknown identity was almost not detectable in the reference strain (Figure 4). It seems that the absence of Gcy1 facilitates the conversion of d-GalUA to a product different from l-GalA.

The knowledge obtained in the current study will be useful for metabolic engineering endeavours in the yeast *S. cerevisiae* in the context of designing biorefinery concepts for pectin-rich residues resulting from the food industry. In fact, there have been several envisaged applications of the yeast *S. cerevisiae* in this context. As detailed in the introduction, it is obvious to equip baker’s yeast with substrate consumption pathways that allow ethanol production from pectin-rich biomass including d-GalUA. However, baker’s yeast has also been considered for biotransformations of d-GalUA to *meso*-galactarate [27] and l-GalA [32,61,64]. Galactarate can be further converted to adipic acid [27,65], and l-GalA is a precursor of l-ascorbic acid (vitamin C) synthesis [66]. A very recent review about the achievements in this field has been provided by Jeong et al. [6]. Depending on the envisaged bioprocess, l-GalA formation from d-GalUA might be either unwanted (if *meso*-galactarate is the product) or supportive (when baker’s yeast is equipped with a pathway in which l-GalA is an intermediate). Respectively, *GCY1* deletion or overexpression might be considered.

## Figures and Tables

**Table 1:** *S. cerevisiae* strains used in this study.

Strain	Genotype, Description	Reference
CEN.PK113-1A	*MAT*α (prototrophic)	Euroscarf
CEN.PK113-1A *UBR2_CBS_*	*MAT*α; *ubr2*::*UBR2_CBS 6412-13A_*	[33]
CEN.PK RE	*MAT*α; *ubr2*::*UBR2_CBS 6412-13A_*; *gut1*::*GUT1_JL1_*	This study *
CEN.PK DHA	*MATα*; *ubr2*::*UBR2_CBS 6412-13A_*; *YGLCτ3*::*P_TEF1_-CjFPS1-T_CYC1_*; *gut1*::*P_TEF1_-Opgdh-T_CYC1_-P_ADH2_-ScDAK1-T_TPS1_*	[34]
CEN.PK DHA *gcy1*Δ	*MATα*; *ubr2*::*UBR2_CBS 6412-13A_*; *YGLCτ3*::*P_TEF1_-CjFPS1-T_CYC1_*; *gut1*::*P_TEF1_-Opgdh-T_CYC1_-P_ADH2_-ScDAK1-T_TPS1_*; *gcy1*::*loxP-ble-loxP*	This study
CEN.PK DHA *gre3*Δ	*MATα*; *ubr2*::*UBR2_CBS 6412-13A_*; *YGLCτ3*::*P_TEF1_-CjFPS1-T_CYC1_*; *gut1*::*P_TEF1_-Opgdh-T_CYC1_-P_ADH2_-ScDAK1-T_TPS1_*; *gre3*::*loxP-ble-loxP*	This study
CEN.PK DHA *ypr1*Δ	*MATα*; *ubr2*::*UBR2_CBS 6412-13A_*; *YGLCτ3*::*P_TEF1_-CjFPS1-T_CYC1_*; *gut1*::*P_TEF1_-Opgdh-T_CYC1_-P_ADH2_-ScDAK1-T_TPS1_*; *ypr1*::*loxP-ble-loxP*	This study

* The maximum specific growth rate of this strain has already been reported in the study of Ho et al. [35], where the strain was referred to as CEN.PK113-1A *GUT1_JL1_ UBR2_CBS_*. The construction of this strain is described in Material and Methods of the current study. *UBR2*: encoding cytoplasmic ubiquitin-protein ligase; *GUT1*: encoding glycerol kinase; *CjFPS1*: encoding aquaglyceroporin from *C. jadinii*; *Opgdh*: encoding glycerol dehydrogenase from *O. parapolymorpha*; *DAK1*: encoding dihydroxyacetone kinase; *GCY1*, *YPR1* and *GRE3*: encoding unspecific aldo-keto reductases.

## Data Availability

Data are available in this article and as Supplementary Material.

## Supplementary Materials

The following are available online at https://www.mdpi.com/article/10.3390/jof7110914/s1, Figure S1: Routes of glycerol catabolism in the glycerol-negative wild-type strain (A) and the two glycerol-positive strains used in this study in order to prove d-galacturonic acid (d-GalUA) conversion to l-galactonate (l-GalA) in synthetic medium and the presence of glycerol (B and C), Figure S2: Comparison of the CEN.PK DHA strain with the reverse engineered CEN.PK strain (CEN.PK RE) in synthetic glycerol medium supplemented with d-GalUA, Table S1: Plasmids used and constructed in this study, Table S2: PCR primers used for constructing and verifying the deletion mutants for *gcy1*Δ, *ypr1*Δ and *gre3*Δ in the *S. cerevisiae* strain CEN.PK DHA, Table S3: PCR primers used for the marker replacement in the 2*µ*-based expression vectors p424GPD and p424GCY1.
