# Supplementary material for: Identification of the Aldo-Keto Reductase Responsible for d-Galacturonic Acid Conversion to l-Galactonate in Saccharomyces cerevisiae
Source: J Fungi (Basel). 2021 Oct 27;7(11):914. doi: 10.3390/jof7110914 (PMC8622349; doi:10.3390/jof7110914)
Supplement: Supplementary file 1 [file jof-07-00914-s001.zip › jof-1356492-supplementary.pdf]

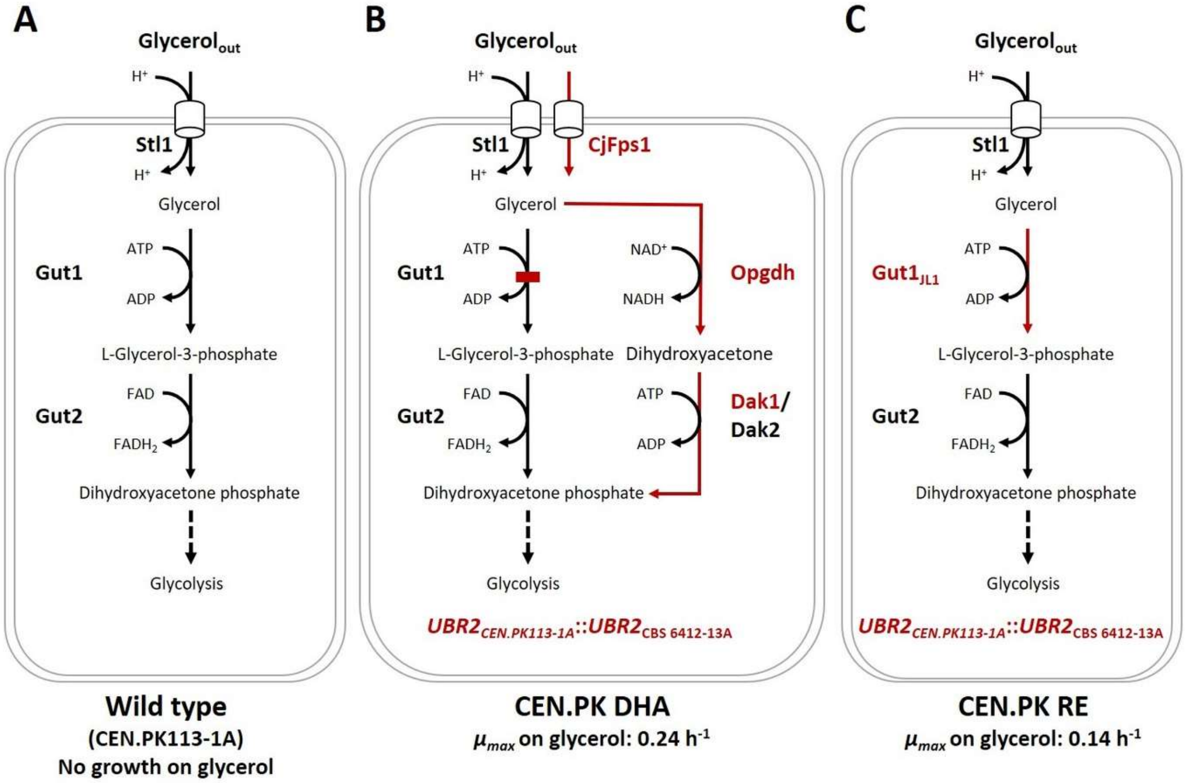

Figure S1: Routes of glycerol catabolism in the glycerol-negative wild-type strain (A) and the two glycerol-positive strains used in this study in order to prove D-galacturonic acid (D-GalUA) conversion to L-galactonate (L-GalA) in synthetic medium and the presence of glycerol (B and C). In the wild-type *S. cerevisiae* strain CEN.PK113-1A (A), glycerol enters the cell via glycerol/H^+ symporter encoded by *STL1*. It is then catabolized via the L-glycerol-3-phosphate (L-G3P) pathway, comprised of a glycerol kinase encoded by *GUT1* and a FAD-dependent L-G3P dehydrogenase encoded by *GUT2*. The resulting intermediate dihydroxyacetone phosphate (DHAP) is channelled into the cell's central carbon metabolism. In the strain CEN.PK DHA (B), the native glycerol catabolic pathway is replaced by the NAD-dependent dihydroxyacetone (DHA) pathway which is composed of a heterologous glycerol dehydrogenase from *Ogataea parapolymorpha* (Opgdh) and the endogenous dihydroxyacetone kinase (Dak1). The endogenous *DAK1* gene was overexpressed in order to allow efficient growth on glycerol [44]. The strain CEN.PK DHA also carries an expression cassette for a heterologous aquaglyceroporin from *Cyberlindnera jadinii* (CjFps1) for improved glycerol uptake [45] and a replacement of the native *UBR2* allele by the respective allele from the natural *S. cerevisiae* isolate CBS 6412-13 [33]. In the reversed-engineered strain CEN.PK RE (C), the endogenous *UBR2* allele was also replaced by *UBR2* from strain CBS 6412-13A and the wild-type CEN.PK *GUT1* allele was replaced by the *GUT1* allele from JL1 [35]. All genetic modifications applied for pathway modifications are shown in red. Stl1: glycerol/H^+ symporter; CjFps1: Fps1 homolog (aquaglyceroporin) from *C. jadinii*; Gut1: glycerol kinase; Gut2: mitochondrial (membrane bound) FAD-dependent L-glycerol-3-phosphate dehydrogenase; Dak1/Dak2: dihydroxyacetone kinase; Opgdh: glycerol dehydrogenase from *O. parapolymorpha*; *UBR2*: encoding cytoplasmic ubiquitin-protein ligase.

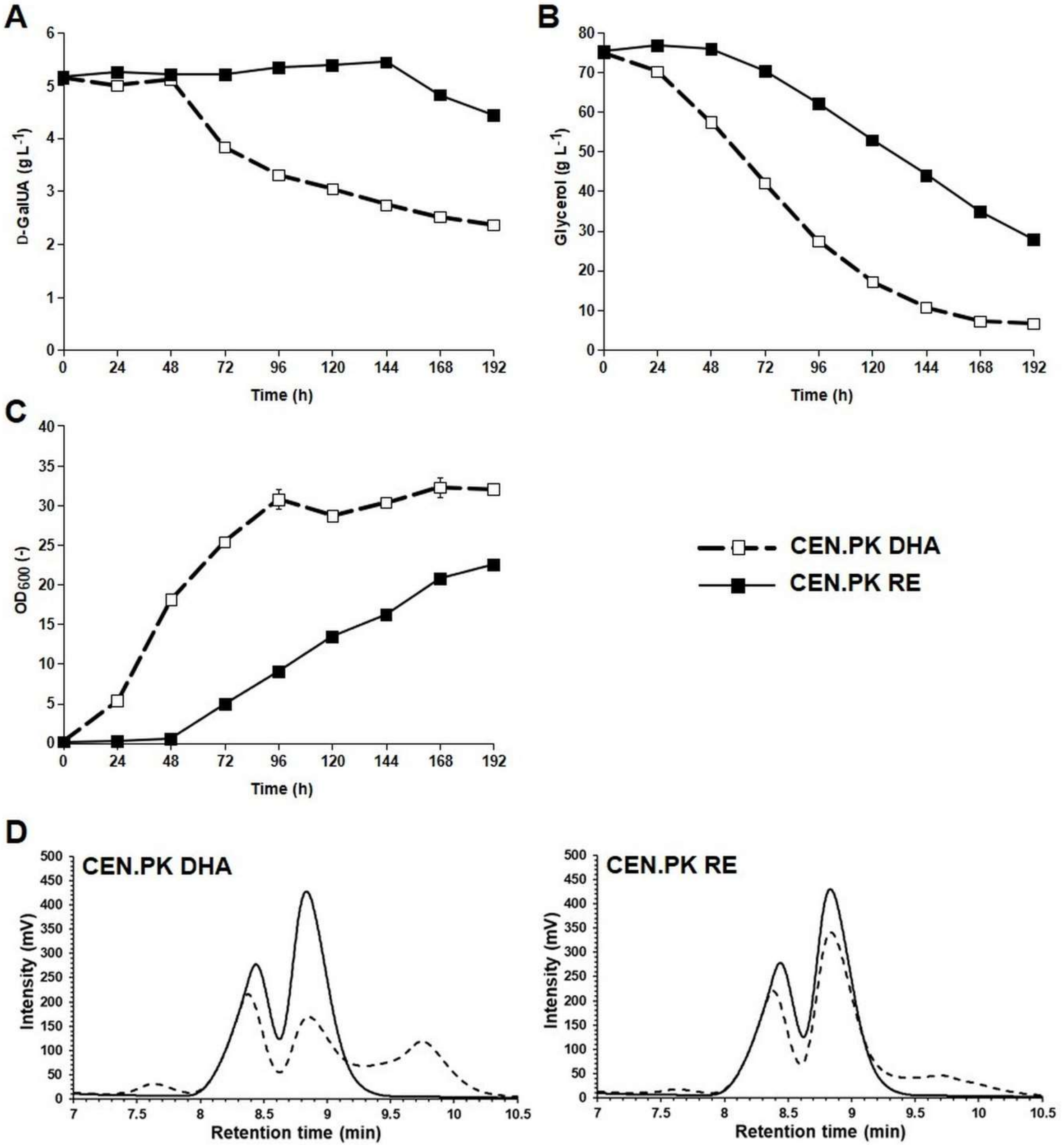

Figure S2: Comparison of the CEN.PK DHA strain with the reverse-engineered CEN.PK strain (CEN.PK RE) in synthetic glycerol medium supplemented with D-GalUA. Cultivations were performed in 500 mL flasks containing 100 mL of synthetic medium with glycerol and D-GalUA with an initial pH of 3 and urea as the source of nitrogen. Samples were taken in 24 h intervals and culture supernatants were analysed by HPLC to follow the consumption of D-GalUA (A) and glycerol (B). Growth was recorded by determining the optical density of the cultures at a wavelength of 600 nm (OD_{600}) (C). All mean values and standard deviations were derived from biological triplicates. (D) HPLC chromatograms of culture supernatants. D-GalUA and L-GalA were detected via the Waters 2412 RI detector. The retention time of D-GalUA was ~8.8 min and the retention time of L-GalA was ~9.7 min. For each strain a sample after 192 hours (dashed line) was compared to a sample from the respective strain that was taken at the beginning of the batch cultivation in shake flasks (straight line).

Table S1: Plasmids used and constructed in this study.

Plasmid name	Relevant characteristics	Reference
pUG66	*loxP-ble-loxP* disruption cassette (conferring phleomycin resistance)	[37]
p424GPD	2μ-based expression vector containing the *GPD* promoter (empty vector)	[41]
p424GCY1	Derivative of p424GPD containing the *GCY1* coding sequence	[40]
p424GPD-ble	Derivative of p424GPD (*TRP1* replaced by the *loxP-ble-loxP* marker)	This study
p424GCY1-ble	Derivative of p424GCY1 (*TRP1* replaced by the *loxP-ble-loxP* marker)	This study

Table S2: PCR primers used for constructing and verifying the deletion mutants for *gcy1*Δ, *ypr1*Δ *and gre3*Δ in the *S. cerevisiae* strain CEN.PK DHA. Sequences that bind to the template sequence are shown in lower case.

	No.	Primer name	Sequence (5'-3')
*gcy1*Δ	1334	GCY1_del_F	GCTAAAATTTGGACAGCTCTCATTACTAAATTAAGATAGAAAACCGccagctgaagcttcgtacgc
	1335	GCY1_del_R	CTACATATTACAATCATACGAGAAACACGCAAAAACAAataggccactagtggatctgata
*ypr1*Δ	1338	YPR1_del_F	ATAATCAGGTCGCAAATATACCCACAGATAATAATCTACCccagctgaagcttcgtacgc
	1339	YPR1_del_R	ACTACAGGTTGCTGGAGGCATTAACTGTAATGTAGCGCGCgccgcataggccactagtggatctg
*gre3*Δ	1336	GRE3_del_F	GGGAAAATACTGTAATATAAATCGTAAAGGAAAATTGGAAATTTTTTAAAGccagctgaagcttcgtacgc
	1337	GRE3_del_R	CATATCGTCGTTGAGTATGGATTTTACTGGCTGGAgccgcataggccactagtggatctg
Verification	194	GCY1-ver-fwd	ccctagagtagaaaccgagc
	195	GCY1-ver-rev	gtcgagcaattggctgac
	1342	YPR1_ver_F	gaattgttacaccacccc
	1343	YPR1_ver_R	gtccgaagtttacgttca
	1340	GRE3_ver_F	cccaaggaaaactcctcagc
	1341	GRE3_ver_R	caaatgattttgggaccgc
	18	ble control fw	ccaacctgccatcacgagat
	19	ble control rv	tcatgagatgcctgcaagca

Table S3: PCR primers used for the marker replacement in the 2*μ*-based expression vectors p424GPD and p424GCY1. Sequences that bind to the template sequence are shown in lower case.

	No.	Primer name	Sequence (5'-3')
Marker replacement	445	pCAS9-LoxP-F	TTACAATTTCCTGATGCGGTATTTTCTCCTTACGCATCTGTGCGGTATTTCACACCGCAT ccagctgaagcttcgtacgc
	446	pCAS9-LoxP-R	GCGCGTTTCGGTGATGACGGTGAAAACCTCTGACACATGCAGCTCCCGGAGACGGTCACA gcataggccactagtggatctg
Verification	448	pCAS9-VER-R	cgtatcacgaggccctttcgt
	18	ble control fw	ccaacctgccatcacgagat
